# Factor-specific generative pattern from large-scale drug-induced gene expression profile

**DOI:** 10.1038/s41598-023-33061-x

**Published:** 2023-04-18

**Authors:** Se Hwan Ahn, Ju Han Kim

**Affiliations:** 1 Department of Biomedical Sciences, Seoul National University Biomedical Informatics (SNUBI), Seoul National University College of Medicine, Seoul, Republic of Korea; 2 Division of Biomedical Informatics, Seoul National University Biomedical Informatics (SNUBI), Seoul National University College of Medicine, Seoul, Republic of Korea

**Keywords:** Drug discovery, Gene expression, Computational biology and bioinformatics, Microarrays

## Abstract

Drug discovery is a complex and interdisciplinary field that requires the identification of potential drug targets for specific diseases. In this study, we present FacPat, a novel approach that identifies the optimal factor-specific pattern explaining the drug-induced gene expression profile. FacPat uses a genetic algorithm based on pattern distance to mine the optimal factor-specific pattern for each gene in the LINCS L1000 dataset. We applied Benjamini–Hochberg correction to control the false discovery rate and identified significant and interpretable factor-specific patterns consisting of 480 genes, 7 chemical compounds, and 38 human cell lines. Using our approach, we identified genes that show context-specific effects related to chemical compounds and/or human cell lines. Furthermore, we performed functional enrichment analysis to characterize biological features. We demonstrate that FacPat can be used to reveal novel relationships among drugs, diseases, and genes.

## Introduction

Identifying interactions between drugs and targets is important for discovering new drug candidates and repurposing existing ones^1^. Traditionally, the interaction between a drug and a target has been identified through clinical observations and biological experiments^2^. However, traditional gene expression profiling measured using microarray is time-consuming and expensive^3^. Owing to the development of modern high-throughput technology, large-scale gene expression profile data have accumulated^4^. These datasets enable the identification of biological mechanisms of drugs, diseases, and genetic factors^5^.

The Library of Network-based Cellular Signatures (LINCS), a program developed by the National Institutes of Health (NIH), generated large-scale perturbation-induced gene expression profiles^6^. The LINCS consortium generated the L1000 dataset measured using a high-throughput gene expression assay called the L1000 assay. Of 12,328 genes, the expression levels of 978 genes, termed landmark genes, were directly measured using the L1000 assay. The remaining 11,350 non-landmark genes were inferred from the computational model with Gene Expression Omnibus (GEO)^7^ data. The L1000 dataset provides large-scale multivariate gene expression signatures comprising thousands of perturbations to over 70 human cell lines under many different experimental conditions. Thus, the L1000 dataset is useful for pharmacogenomic research, and many different computational methods with the L1000 dataset have been proposed for predicting the mechanism of actions of drugs or repurposing the known ones^8–10^.

Although there are numerous biological features in large-scale multivariate datasets, such as the L1000 data, only a few are important^11^. The L1000 dataset provides more than one million drug-induced gene expression profiles obtained under various experimental conditions, including drugs, doses, cell lines, and time points. Identifying differentially expressed genes (DEGs) between perturbation and control conditions has facilitated the discovery of significant biological features from large-scale multivariate drug-induced gene expression profiles. DEGs are commonly identified using conventional statistical methods, such as analysis of variance (ANOVA) and multivariate analysis of variance (MANOVA); however, these methods are limited as they require a sufficient number of replicate experiments for accurate identification^12^. Additionally, analysis of the distribution of replicate experiments in the L1000 dataset revealed that approximately 98% of the dataset was measured from samples with one to eight replicate experiments, with most samples having three replicates^13^. Therefore, the development of novel approaches and methods that can effectively analyze the L1000 dataset is required.

**Figure 1:**
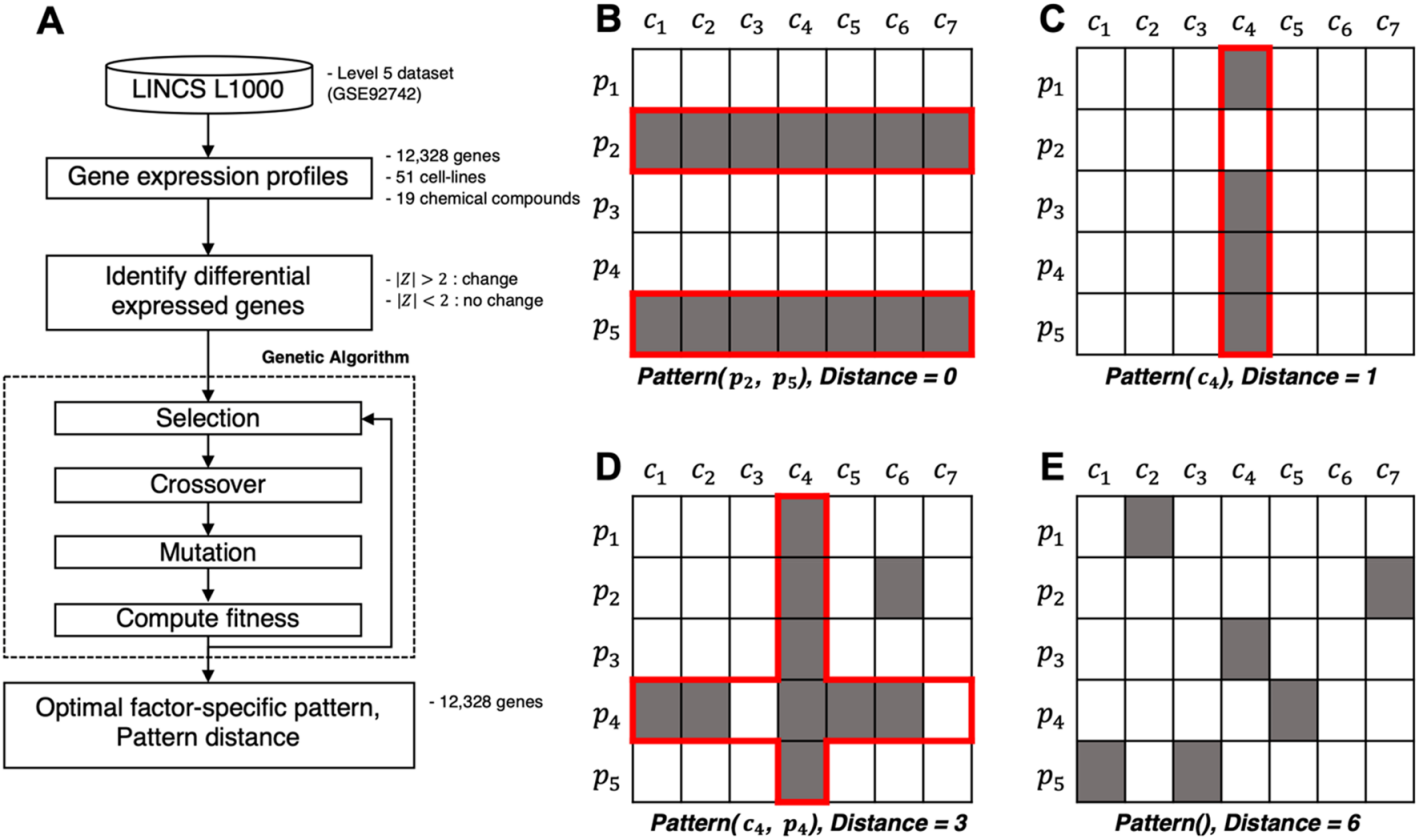
(**A**) Workflow schema of FacPat. For our analysis, the gene expression profiles of 51 cell lines treated with 19 chemical compounds from the L1000 dataset were constructed. The threshold $\left|Z\right|$ > 2 was used to identify differentially expressed genes (DEGs) in the expression profile. The optimal biological factor-specific patterns are identified by a genetic algorithm. (**B**–**D**) The optimal factor-specific pattern (red line) and pattern distance (d) from the expression profile using a genetic algorithm. The gray color shows identified DEGs. (**B**) The ${{{p}}}_{2}$- and ${{{p}}}_{5}$-specific pattern (d = 0). (**C**) The ${{{c}}}_{4}$-specific pattern (d = 1). (**D**) The ${{{c}}}_{4}$- and ${{{p}}}_{4}$-specific pattern (d = 3). (**E**) The null pattern (d = 6). In binary space, pattern distance is defined as the count of mismatches. ${{{c}}}_{{{i}}}$, the *i*th cell line; ${{{p}}}_{{{i}}}$, the *i*th perturbagen.

Data-mining technology facilitates the extraction of useful information from large-scale data^14^. The present study aimed to identify the optimal biological factors that describe the expression profile using a method that mines the gene expression patterns. We propose a novel method named FacPat that can identify key biological factor-specific patterns among chemical compounds, human cell lines, and genes using perturbation-induced gene expression signatures from the L1000 dataset (Fig. 1A). We first constructed an expression profile for every 12,328 genes comprising the gene expression signatures of 51 human cell lines treated with 19 chemical compounds. We assumed that the expression profile was combined with noise and an underlying factor-specific pattern. To quantify the impact of noise, we measured the pattern distance by counting the number of mismatch elements between the observed expression profile and factor-specific pattern. Therefore, the optimal factor-specific pattern had the closest pattern distance between the factor-specific pattern and the observed expression profile. We then used a genetic algorithm to determine the optimal factor-specific pattern for each observed expression profile (Fig. 1B–E) and generated the distribution of pattern distances for each observed expression profile to address multiple testing corrections. Finally, we identified significant and directly interpretable biological factor-specific patterns in the L1000 dataset. FacPat identified the relationships among chemical compounds, human cell lines, and genes that describe the expression profiles. The unique advantage of FacPat lies in its ability to identify these significant patterns without the need for sufficient replications, thereby overcoming the limitations of traditional statistical methods, such as ANOVA and MANOVA.

## Results

### Overview of FacPat

In the present study, we developed a novel approach called FacPat for identifying significant biological key factor-specific patterns for each gene in the L1000 dataset. For our analysis, we constructed a complete expression profile for each gene using expression signatures of 51 cell lines treated with 19 chemical compounds at the 6-h time point (Tables 1 and 2).

**Table 1:** List of 51 cell lines by primary sites.

Primary site	Cell line
Bone	A673
Breast	MCF7
Endometrium	HEC108, JHUEM2, SNGM
Hematopoietic and lymphoid tissue	NOMO1, PL21, SKM1, THP1, U937, WSUDLCL2
Kidney	HA1E
Large intestine	CL34, HCT116, HT115, HT29, LOVO, MDST8, NCIH508, RKO, SNU1040, SNUC4, SNUC5, SW480, SW620, SW948
Liver	HEPG2
Lung	A549, CORL23, DV90, NCIH1299, HCC15, HCC515, NCIH1694, NCIH1836, NCIH2073, NCIH596, SKLU1, T3M10
Ovary	COV644, EFO27, OV7, RMGI, RMUGS, TYKNU
Prostate	PC3, VCAP
Stomach	AGS
Skin	A375, SKMEL1, SKMEL28

**Table 2:** List of 19 chemical compounds.

PubChem CID	Name	Dosage	Mechanism of action
3413	Forskolin Racemate	10 μM	Adenylyl cyclase activator
441294584	Alda-1	40 μM	Aldehyde dehydrogenase activator
24857885	PTP1B-IN-3	10 μM	AMP-activated protein kinase activator
200	AICA-ribonucleotide	10 μM	AMPK activator
135421197	PAC-1	10 μM	Caspase activator
44197249	BRD-K30064966	10 μM	Caspase activator
6376322	Trichostatin-A (TSA)	10 μM	Histone deacetylase inhibitor
9886086	Ro-28-1675	160 μM	Glucokinase activator
638278	Isoliquiritigenin	10 μM	Guanylate cyclase activator
4201	Minoxidil	10 μM	ATP-sensitive potassium channels activator
6603728	BAY-K-8644-(S)-(−)	10 μM	L-type calcium channel activator
761523	m-3M3FBS	80 μM	Phospholipase activator
442042	Ingenol 3,20-dibenzoate (IDB)	10 μM	Protein Kinase C activator
4792	Phorbol-12-myristate-13-acetate (PMA)	10 μM	Protein Kinase C activator
10474339	BMS-191011	10 μM	Potassium channel activator
60138087	M2-PK-activator	90 μM	Pyruvate kinase isozyme activator
445154	Resveratrol	10 μM	Sirtuin activator
44240264	SRT-1720	10 μM	Sirtuin activator
237	Mepacrine	10 μM	TP53 activator

**Figure 2:**
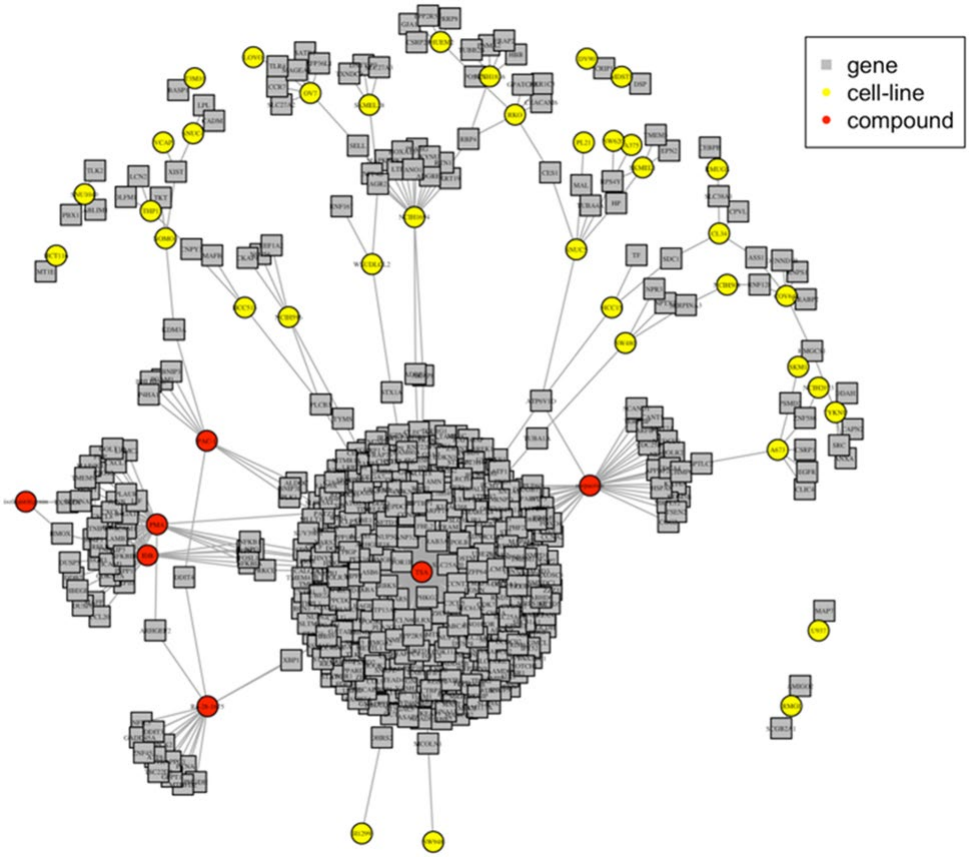
Gene, cell line, and chemical compound association network graph visualized using the igraph R package. The network visualizes the significant factor-specific patterns (FDR < 0.05) obtained from FacPat, representing the associations among 480 genes, 38 cell lines, and 7 chemical compounds. The nodes in the graph represent individual genes, cell lines, and chemical compounds, while the edge connections indicate their associations.

To determine differential expression, we dichotomized the expression signatures using a threshold of |Z|> 2.0, which indicates significantly altered gene expression signatures compared to the control. The optimal factor-specific pattern was determined using a genetic algorithm from the observed expression profiles of all 12,328 genes based on the pattern distance (Fig. 1A). Of these genes, 480 were judged significant with an false discovery rate (FDR) of < 5% (Supplementary Table 1 and Fig. 2). In Fig. 2, we show the significant and interpretable interactions for 480 genes, 7 chemical compounds, and 38 cell lines identified from the L1000 dataset (FDR < 0.05). A total of 383 genes showed only chemical compound-specific effects, 86 genes showed only cell-specific effects, and 11 genes showed both chemical compound- and cell-specific effects.

### Evaluation

We compared our results with the Comparative Toxicogenomics Databases (CTD)^15^ to determine the extent of overlap between our findings and previously reported relationships. The CTD is a comprehensive public resource that curates data on the relationships among chemicals, genes, and diseases. Our analysis revealed that 56.04% (269 out of 480) of the genes that we identified as significant were previously reported in the CTD. Notably, among the 11 genes that exhibited both cell line- and chemical compound-specific effects, 8 genes were previously reported in the CTD.

### Characterizing biological features through enrichment analysis

**Figure 3:**
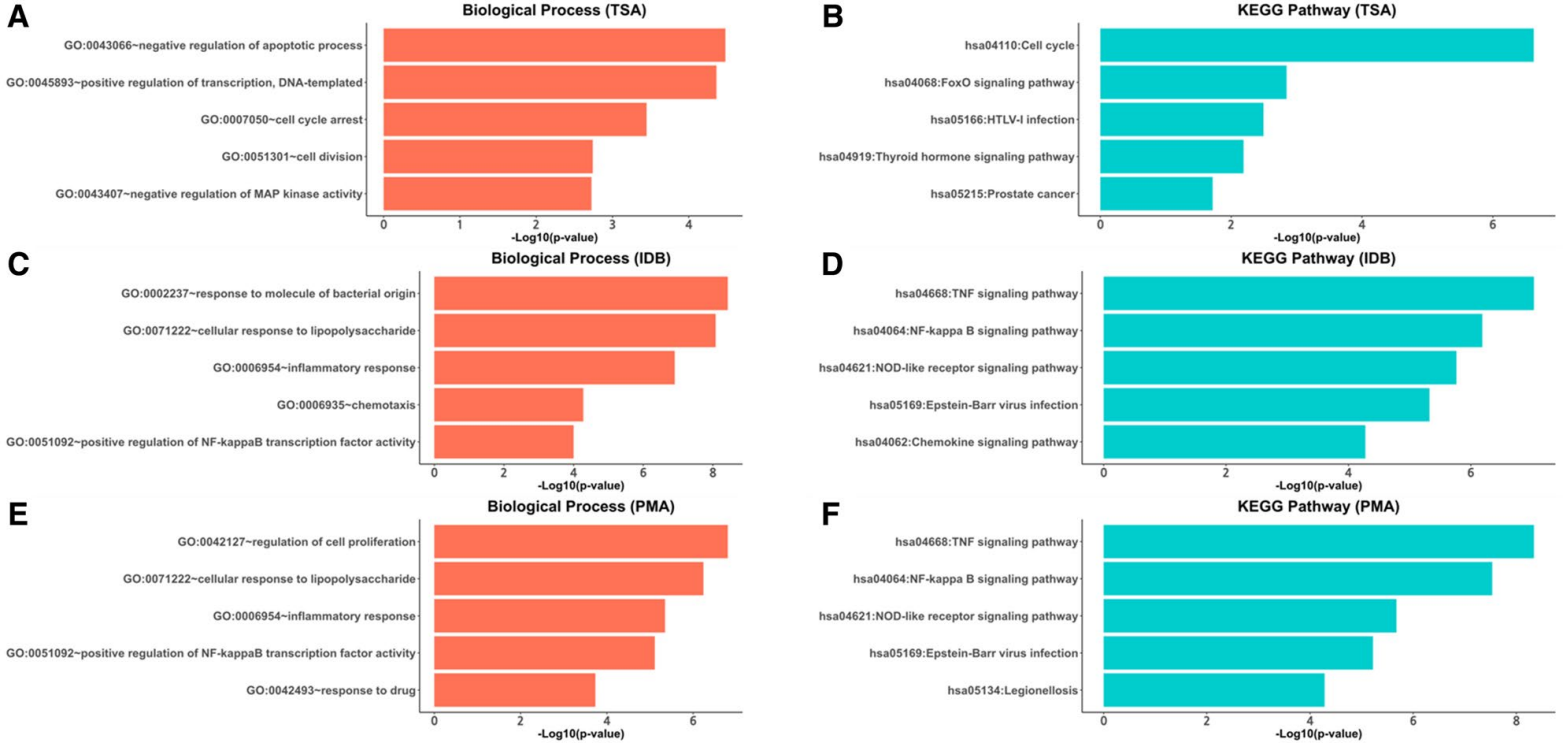
Results of Gene Ontology (GO) term and Kyoto Encyclopedia of Genes and Genomes (KEGG) enrichment analysis for each TSA, IDB, and PMA-specific gene. Due to a large number of significant results, we visualized the top five significant terms based on statistical significance (p < 0.05) for each category. (**A**,**B**) Enriched GO terms of biological processes and KEGG pathways for TSA-specific genes, respectively. (**C**,**D**) Enriched GO terms of biological processes and KEGG pathways for IDB-specific genes, respectively. (**E**,**F**) Enriched GO terms of biological processes and KEGG pathways for PMA-specific genes, respectively. *TSA* Trichostatin-A, *IDB* Ingenol 3,20-dibenzoate, *PMA* Phorbol-12-myristate-13-acetate.

We conducted functional enrichment analysis to identify the biological features of genes that are specific to certain chemical compounds and/or cell types. Our findings revealed significant results for genes related to trichostatin-A (TSA), ingenol 3,20-dibenzoate (IDB), and phorbol-12-myristate-13-acetate (PMA) in the Biological Process (BP) and KEGG pathways (Fig. 3) but not for genes showing only cell-specific effects.

We found that 66.5% (319 out of 480) of genes were specifically associated with TSA, which was initially isolated from *Streptomyces hygroscopicus*^16^. Functional enrichment analysis highlighted that these TSA-specific genes are significantly associated with the negative regulation of the apoptotic process (GO:0043066) and the cell cycle (hsa04110) (Fig. 3A,B). These findings align with the reported anticancer properties of TSA, which functions as a histone deacetylase (HDAC) inhibitor, leading to cell apoptosis and growth arrest^17^. TSA causes hyperacetylation of histones, thereby altering gene expression patterns and ultimately resulting in cell cycle arrest, induction of apoptosis, and inhibition of tumor cell proliferation^17^.

IDB exhibits various biological activities, including anti-inflammatory and anticancer effects^18,19^; therefore, further understanding of the precise mechanism of action of IDB is crucial for its potential therapeutic applications. Despite ongoing research and numerous studies, the precise mechanism of action of IDB is yet to be fully elucidated^20,21^. We found that 6.7% (32 out of 480) of the significant genes exhibited IDB-specific effects (Fig. 2). As shown in Fig. 3C,D, IDB-specific genes were significantly enriched in the inflammatory response (GO:0006954), TNF signaling pathway (hsa04668), NF-κB signaling pathway (hsa04064), and NOD-like receptor signaling pathway (hsa04621). These results are consistent with previously reported findings, where IDB has been shown to modulate inflammation and immune responses through its effects on signaling pathways, such as NF-κB and TNF^22^. Overall, our findings are consistent with previous studies on the mechanism of action of IDB, highlighting its potential as a therapeutic agent targeting inflammation and immune-related pathways.

In addition, we found that 7.5% (36 out of 480) of the significant genes exhibited a PMA-specific effect (Fig. 2). As shown in Fig. 3E,F, PMA-specific genes were also significantly enriched in the inflammatory response (GO:0006954), TNF signaling pathway (hsa04668), NF-κB signaling pathway (hsa04064), and NOD-like receptor signaling pathway (hsa04621). Furthermore, we identified 24 genes that were associated with both IDB and PMA, and these genes also demonstrated significant enrichment in the inflammatory response (GO:0006954) and the NF-κB signaling pathway (hsa04064). Our findings suggest that IDB and PMA may exert their biological effects through common mechanisms, particularly in the modulation of inflammation and immune responses. This result is also supported by their shared mechanism to activate PKC, a key enzyme involved in signal transduction and the regulation of various cellular processes^18,23^.

### Both chemical compound- and cell-specific genes

**Figure 4:**
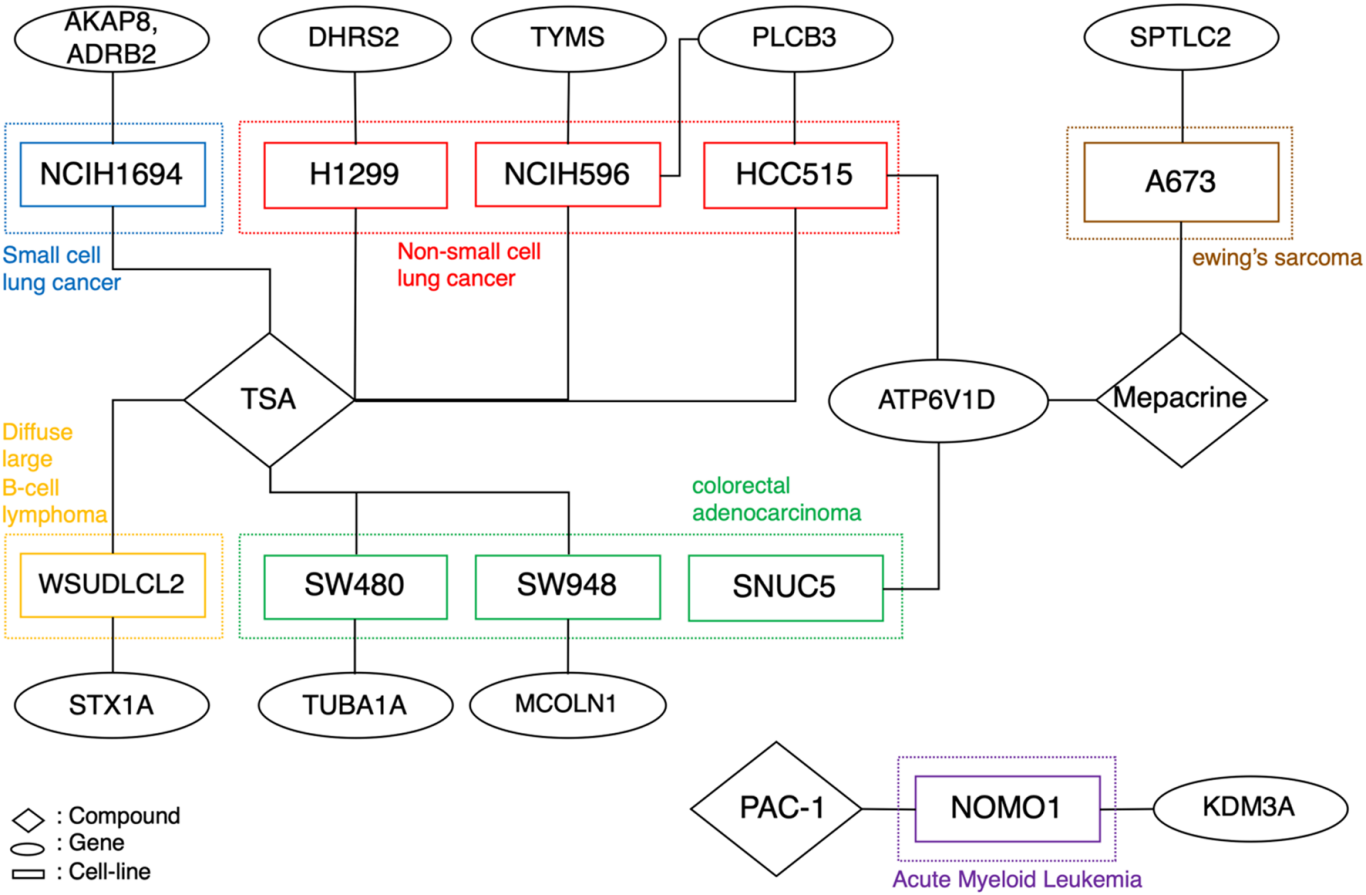
Cell line and chemical compound-specific patterns. The significant factor-specific patterns exhibiting both chemical compound- and cell-specific effects are visualized.

Subsequently, we focused on 11 genes that exhibited both chemical compound- and cell-specific effects. The significant optimal factor-specific patterns for the 11 genes are shown in Fig. 4. AKAP8 and ADRB2 showed specific effects in both TSA- and small-cell lung cancer (SCLC) cell lines, NCIH1694. DHRS2, TYMS, PLCB3, and ATP6V1D showed both TSA- and non-small-cell lung cancer (NSCLC) cell line-specific effects. ATP6V1D was also associated with mepacrine and SNUC5, the only gene associated with dual-chemical compounds and cell lines. SPTLC2 exhibited both mepacrine and A673-specific effects. KDM3A showed both PAC-1- and NOMO1-specific effects. MCOLN1 and TUBA1A were associated with both TSA- and colorectal cancer cell line-specific effects. Moreover, STX1A exhibited both TSA- and WSUDLCL2-specific effects.

## Discussion

In this study, we developed a novel approach, FacPat, for identifying context-specific associations among genes, chemical compounds, and human cell lines, using gene expression profiles from the LINCS L1000 dataset. FacPat is based on a genetic algorithm and uses pattern distance to determine the optimal factor-specific pattern from observed gene expression profiles. Using this approach, we identified 480 significant genes specifically associated with chemical compounds and/or cell lines at an FDR < 0.05. We also performed functional enrichment analysis to identify biological processes and pathways affected by the identified genes. Our results provide insights into the different context-specific effects of genes, which are potential targets for disease treatment.

Our approach has several novel aspects. First, we focused on identifying genes that are specifically associated with chemical compounds and/or human cell lines, which can facilitate the identification of potential drug targets for specific diseases. Second, we used a genetic algorithm to identify the optimal factor-specific pattern, which allowed for the identification of subtle but important differences in gene expression patterns. Third, we used pattern distance to quantify the impact of noise and determine the closest factor-specific pattern. Finally, we performed functional enrichment analysis to further explore the biological processes and pathways influenced by the identified genes.

Our results revealed that all significant genes can be interpreted as three context-specific effects. The first effect is associated with genes that display only chemical compound-specific effects, which suggests their involvement in chemical interactions across different diseases. The second effect pertains to genes that display cell line-specific effects, indicating their association with disease-specific molecular mechanisms, irrespective of the chemical compound treatment. The third effect suggests that these genes, which are specific to both chemical compounds and cell lines, can be targeted by chemical compounds for treating specific diseases. Moreover, we identified several genes that are potential targets for therapeutic interventions in various cancers. Specifically, two genes, AKAP8 and ADRB2, were associated with SCLC and trichostatin-A (TSA). TSA is an anticancer drug that inhibits the growth of lung cancer cells through histone hyperacetylation, and AKAP8 is involved in DNA replication and condensation during the cell cycle^24–27^. ADRB2 is associated with the beta-adrenergic receptor ($\beta$-AR), whose activation promotes the progression of lung cancer^28^. Several studies have been conducted to elucidate the mechanism of action of $\beta$-ARs in lung cancer. However, further studies investigating ADRB2 as a candidate target gene for TSA in NSCLC are required.

In the present study, we identified four genes, ATP6V1D, TYMS, PLCB3, and DHRS2, that are associated with both TSA and NSCLC. ATP6V1D encodes a vacuolar ATPase (V-ATPase), and in NSCLC, chemotherapy drug resistance is associated with the expression of V-ATPase^29^. TYMS is a common target gene of HDAC inhibitors and is suppressed by HDAC inhibition^30^. PLCB3 is associated with poor overall survival of patients with NSCLC and poor prognosis of adenocarcinoma^31^; however, the interaction between PLCB3 and TSA has not yet been discovered. DHRS2 is associated with various functions, such as cell proliferation and migration, in many different cancers^32^. In our study, we found that it may be a novel target of TSA in NSCLC. These findings suggest that genes showing both TSA- and NSCLC-specific effects may be potential targets of TSA in NSCLC.

In addition, we found that another gene, SPTLC2, was associated with both mepacrine and the human Ewing's sarcoma cell line, A673. Mepacrine promotes apoptotic signaling through several pathways, including inducing p53^33^. Small-molecule p53 activators, such as actinomycin D, are being considered as potential treatments for Ewing's sarcoma^34^. Therefore, SPTLC2 may be a novel mepacrine target for treating human Ewing’s sarcoma. We also found that KDM3A is related to both PAC-1 and the human acute myeloid leukemia (AML) cell line NOMO1. The role of KDM3A in AML has not yet been fully elucidated; however, it is known to promote the growth of many solid tumors^35^. PAC-1 increases the concentration of caspase-3 and has been studied extensively as a strategy for treating many cancers, including leukemia^36^. These findings suggest that KDM3A is a potential target for the treatment of leukemia.

Furthermore, we identified two genes, TUBA1A and MCOLN1, which are associated with TSA and colorectal adenocarcinoma. TUBA1A is one of the three α-tubulin genes, and TSA induces α-tubulin acetylation, which effectively inhibits HDAC6^37^. In colon cancer, HDAC6 expression is high and associated with poor prognosis^38^. Therefore, TUBA1A may act as a potential target when TSA is used to treat colon cancer. MCOLN1, a member of the mucolipin family of transient receptor potential channels (TRPMLs), is significantly differentially expressed among colon cancer cells^39^. In this study, we found that MCOLN1 is a novel target of TSA for the treatment of colon cancer. Forever, further studies are required to identify the biological processes of MCOLN1 and TSA in colon cancer.

Our approach can be used to discover novel drug targets for disease treatment from large-scale drug-induced expression profiles. We focused on two biological factors, human cell lines, and chemical compounds. However, they can also be extended to other biological factors. For example, it can be applied to determine the concentration of a drug to identify dose-specific effects. Additionally, it is scalable to an N-dimensional matrix rather than a two-dimensional matrix, allowing for the identification of higher-order interactions of biological factors. Moreover, we computed the pattern distance between the observed expression profile and the biological factor-specific pattern by counting the mismatch elements. However, it is also possible to use other methods to compute pattern distances. In summary, we believe that our FacPat approach is valuable for uncovering biologically relevant patterns, and it has the potential to be applied to other large-scale datasets, further advancing our understanding of drug action and disease mechanisms.

Our study has some limitations. First, when there are several optimal factor-specific patterns for each gene that are not null patterns, one of them is randomly selected. In addition, we only focused on the optimal biological factor-specific pattern that describes the expression profiles of differentially expressed signatures; however, patterns with the closest pattern distance and the other patterns were also statistically significant.

In conclusion, our approach has the potential to identify novel drug targets for disease treatment from large-scale gene expression datasets. Our findings contribute to the growing body of research on the identification of context-specific patterns, which will improve our understanding of disease pathogenesis and facilitate the development of more effective treatments.

## Methods

### Drug-induced gene expression data from the LINCS dataset

In the L1000 dataset, there are approximately 1.3 million gene expression profiles that are perturbed in over 70 human cell lines with 16,425 perturbations induced by chemical compounds (e.g., drugs and small molecules) and 5806 genetic perturbations (e.g., over-expression and single-gene knockdown) under various experimental conditions (e.g., dose and time point)^40,41^. The L1000 dataset contains five preprocessing steps and provides the dataset for each step. In summary, the level 1 data consist of raw fluorescent intensity values measured using Luminex scanners, level 2 is the deconvolution step from the measured fluorescent intensity values of 978 landmark genes, level 3 is the inference step for 11,350 non-landmark genes based on the normalized values for the 978 landmark genes, level 4 data consist of z-scores for each gene based on level 3, and level 5 data consist of replicate collapsed z-score signatures based on level 4 by moderated z-scores (MODZ) procedure^6^. All levels of L1000 datasets are deposited into the GEO database and are available for download. Therefore, we downloaded L1000 level 5 data (GSE92742) from the GEO database.

Although the L1000 dataset is a large-scale dataset, most of the data are focused on only nine core cell lines: A375, A549, HA1E, HCC515, HT29, HEPG2, MCF7, PC3, and VCAP^13^. With these nine core cell lines, all the data in Touchstone, the reference dataset of L1000, was generated. For our analysis, we selected experimental conditions to create a complete expression profile without missing values from the large-scale L1000 dataset. Finally, we constructed a complete expression profile for each of the 12,328 gene expression signatures of 51 cell lines treated with 19 chemical compounds at the 6-h time point (Tables 1 and 2).

### Mining factor-specific pattern algorithm

We hypothesized that the observed expression profile would be combined with noise- and an underlying factor-specific pattern. To quantify the impact of noise, we calculated the pattern distance by counting the number of mismatched elements between the factor-specific pattern and the observed expression profile. Pattern distance was equivalent to the number of mismatches when the expression signature was dichotomized into one (significantly changed) or zero (unchanged). In a two-dimensional matrix, the pattern distance between the observed expression profile (${E}_{ij}$) and factor-specific pattern (${E}_{ij}^{\mathrm{^{\prime}}}$) is defined as $\sum \left|{E}_{ij}-{E}_{ij}^{\mathrm{^{\prime}}}\right|$.

The optimal factor-specific pattern was defined as the closest pattern distance. We applied a genetic algorithm^42^ to identify the optimal factor-specific pattern from the observed expression profile. Through the selection, crossover, mutation, and mating steps, the optimal factor-specific pattern was determined (Fig. 1A).

As shown in Fig. 1B, the optimal factor-specific pattern matches the observed expression profile perfectly, resulting in a pattern distance of zero. Figure 1C shows an expression profile that has a single mismatch with the optimal factor-specific pattern, Pattern (${c}_{4}$), resulting in a distance of 1. Similarly, Fig. 1D depicts an expression profile that has three mismatches with the optimal factor-specific pattern, Pattern (${c}_{4}$,${p}_{4}$), resulting in a distance of 3. When the optimal factor-specific pattern was not specific to any biological factor, we defined it as a null pattern (Fig. 1E).

Because we scored the pattern distance for each gene simultaneously, we applied Benjamini–Hochberg (BH)^43^ correction to control the FDR. To estimate the FDR, we shuffled the observed expression profiles for each group. A group was defined as having the same number of significant elements in the observed expression profile. We defined ${D}_{n}$ as the pattern distance of the observed expression profile, where n is the number of significant elements. Therefore, the pattern distances of the permuted expression profiles can be represented ${D}_{perm}(n)=\{{D}_{n}^{{perm}_{1}},{D}_{n}^{{perm}_{2}},\dots ,{D}_{n}^{{perm}_{L}}\}$, for L = 10,000. From ${D}_{perm}(n)$, we estimated the p-values using:

1 $${P}_{d,n}=Pr\left({D}_{n}\ge {D}_{n}^{perm}\right)=\frac{{\prod }_{k=1}^{L}I\left({D}_{n}\ge {D}_{n}^{{perm}_{k}}\right)}{L}$$

Using Eq. (1), we calculated p-values for each observed expression profile. We then converted p-values into q-values to control the FDR using the BH method^42^. Finally, significant factor-specific patterns were obtained at the 5% significance level.

The association network among genes, cell lines, and chemical compounds from significant factor-specific patterns was visualized using the R igraph software package^44^.

### Functional enrichment analysis

Furthermore, we performed Gene Ontology (GO) analysis using the Database for Annotation, Visualization, and Integrated Discovery (DAVID v6.8)^45,46^ for genes that showed identical significant context-specific patterns. Functional annotations for biological processes (BP) and Kyoto Encyclopedia of Genes and Genomes (KEGG) pathways^47–49^ were used to perform enrichment analysis. The significant results of the enrichment analysis (p < 0.05) were visualized with the R ggplot2 software package^50^.

## Supplementary Information

Supplementary Table 1.

## Data Availability

We used an open-access L1000 dataset from clue.io (https://clue.io). The L1000 dataset was downloaded from the NCBI GEO (accession no.GSE92742).

## Code availability

The code is publicly available on Github (https://github.com/sehwanahn/FacPat).
